# Adult Height and Risk of Colorectal Cancer: A Pooled Analysis of 10 Population-based Cohort Studies in Japan

**DOI:** 10.2188/jea.JE20220289

**Published:** 2024-02-05

**Authors:** Rachana Manandhar Shrestha, Tetsuya Mizoue, Zobida Islam, Yukino Kawakatsu, Hidemi Ito, Keiko Wada, Chisato Nagata, Ling Zha, Tetsuhisa Kitamura, Ritsu Sakata, Takashi Kimura, Yumi Sugawara, Ichiro Tsuji, Ren Sato, Norie Sawada, Shoichiro Tsugane, Yingsong Lin, Isao Oze, Sarah Krull Abe, Manami Inoue

**Affiliations:** 1 Department of Epidemiology and Prevention, Center for Clinical Sciences, National Center for Global Health and Medicine, Tokyo, Japan; 2 Division of Cancer Epidemiology and Prevention, Aichi Cancer Center, Nagoya, Japan; 3 Division of Descriptive Cancer Epidemiology, Nagoya University Graduate School of Medicine, Nagoya, Japan; 4 Division of Cancer Information and Control, Aichi Cancer Center, Nagoya, Japan; 5 Department of Epidemiology and Preventive Medicine, Gifu University Graduate School of Medicine, Gifu, Japan; 6 Division of Environmental Medicine and Population Sciences, Department of Social and Environmental Medicine, Osaka University Graduate School of Medicine, Osaka, Japan; 7 Department of Epidemiology, Radiation Effects Research Foundation, Hiroshima, Japan; 8 Department of Public Health, Hokkaido University Graduate School of Medicine, Sapporo, Japan; 9 Division of Epidemiology, Department of Health Informatics and Public Health, Tohoku University School of Public Health, Graduate School of Medicine, Sendai, Japan; 10 Division of Cohort Research, National Cancer Center Institute for Cancer Control, Tokyo, Japan; 11 Department of Public Health, School of Medicine, Aichi Medical University School of Medicine, Aichi, Japan; 12 Division of Prevention, National Cancer Center Institute for Cancer Control, Tokyo, Japan

**Keywords:** colorectal cancer, colon cancer, rectal cancer, adult height, pooled analysis, Japan

## Abstract

**Background:**

While tall stature has been linked to an increase in the risk of colorectal cancer (CRC), its association with cancer in the colorectum and its subsites remains unclear among Asians.

**Methods:**

We conducted a pooled analysis of 10 population-based cohort studies among adults in Japan. Each study estimated hazard ratios (HRs) and 95% confidence intervals (CIs) for CRC incidence associated with adult height were estimated using Cox proportional hazards regression with adjustment of the same set of covariates were then pooled to estimate summary HRs incidence using random-effect models.

**Results:**

We identified 9,470 CRC incidences among 390,063 participants during 5,672,930 person-years of follow-up. Men and women with tall stature had a higher risk of CRC and colon cancer. HRs for CRC, colon cancer, and distal colon cancer for the highest versus lowest height categories were 1.23 (95% CI, 1.07–1.40), 1.22 (95% CI, 1.09–1.36), and 1.27 (95% CI, 1.08–1.49), respectively, in men and 1.21 (95% CI, 1.09–1.35), 1.23 (95% CI, 1.08–1.40), and 1.35 (95% CI, 1.003–1.81), respectively, in women. The association with proximal colon cancer and rectal cancer was less evident in both sexes.

**Conclusion:**

This pooled analysis confirms the link between tall stature and a higher risk of CRC and colon cancer (especially distal colon) among the Japanese and adds evidence to support the use of adult height to identify those at a higher risk of CRC.

## INTRODUCTION

Colorectal cancer (CRC) is among the most common cancers worldwide.^1^ According to GLOBOCAN 2020, it is the third most commonly diagnosed cancer (10%) and the second leading cause of cancer-related mortality (9.4%).^2^ In Japan, after the second World War, CRC has increased considerably, and it was estimated to be the third-most common cancer in men (15%) and the second-most common in women (16%) and the second leading cause of cancer-related death in men (13%) and the first in women (16%) in 2020.^3^

Adult height, which is determined by genetic and environmental factors (especially childhood nutrition and socioeconomic status) from the time of conception to early adulthood, has been linked to an increase in the risk of many forms of cancer, including CRC.^4^^,^^5^ For CRC, there is firm evidence from meta-analyses by Zhou et al,^6^ Song et al^7^ and Khankari et al^8^ that support the association of tall stature with higher risk of CRC in both men and women, and the World Cancer Research Fund and the American Institute for Cancer Research judged that the association is “convincing”.^9^

The epidemiological data for this association largely depend on those obtained from Caucasians,^6^^–^^8^ while the evidence is limited among the Asian populations, including Japanese, who are on average shorter than Caucasians.^10^ Based on published data from five Japanese studies, we previously did a meta-analysis and found an increased risk of overall CRC and colon cancer but not rectal cancer.^11^ This finding is compatible with the chronological change of each cancer in Japan; colon cancer has shown a much greater increase than rectal cancer after World War II.^12^ That meta-analysis, however, included five studies reporting data for colon cancer but only two studies on rectal cancer. More data, especially those on rectal cancer, are required to confirm whether the association with adult height differs between colon cancer and rectal cancer. Further, no Japanese study reported the association with cancer in the subsites of the colon. Additionally, the results of a meta-analysis based on published data are limited due to the differences in covariates adjusted and the definition of height category across original studies.

To address these issues, we performed a pooled analysis to determine the association between adult height and the risk of CRC and its subsites among 499,585 participants from 10 large population-based cohort studies in Japan using the same exposure category and adjusting for the same set of covariates across the studies.

## METHODS

### Study populations

This study was done within the pooling project by the Research Group for the Development and Evaluation of Cancer Prevention Strategies in Japan using original data from cohort studies. This project aimed to examine the association between lifestyle and cancer risk among the Japanese population. To maintain high quality and comparability of data, the following criteria for the inclusion of cohorts were defined for the present analysis: (1) population-based cohort studies conducted in Japan; (2) studies initiated between the mid-1980s and mid-1990s; (3) studies with more than 30,000 participants; (4) studies that obtained information on anthropometric measurements, including adult-attained height, at baseline; and (5) studies that followed for incidence of CRC. We included 10 cohorts that met these criteria: Japan Public Health Center-based Prospective Study I (JPHC-I),^13^ Japan Public Health Center-based Prospective Study II (JPHC-II),^13^ Japan Collaborative Cohort Study (JACC),^14^ The Miyagi Cohort Study (MIYAGI),^15^ the Ohsaki National Health Insurance Cohort Study (OHSAKI),^16^ Takayama Cohort Study (TAKAYAMA),^17^ The Three-Prefecture Study – Miyagi portion (3-pref MIYAGI),^18^ The Three-Prefecture Study – Aichi portion (3-pref AICHI),^18^ The Three-Prefecture Study – Osaka portion (3-pref OSAKA)^18^ and Life Span Study (LSS).^19^ Participants with missing data on age, sex, and region; invalid or missing information on height; and a history of any cancer at baseline were excluded from the study. All the studies were approved by the relevant institutional ethical review board. Table 1 presents the selected characteristics of the cohort studies.

**Table 1.** Characteristics of the cohort studies in the present pooled analysis

Study	Population	Age at baseline, years	Year of baseline survey	Population size	Response rate (%) of the baseline questionnaire	Method of follow-up	For the present pooled analysis						

							Age, years	Last follow- up time	mean follow up period, years	Size of the cohort		Number of colorectal cancer cases	

										Men	Women	Men	Women
JPHC I	Japanese residents of 5 public health center areas in Japan	40–59	1990	61,595	82%	Cancer registry and death certificate	40–59	2013	20.6	20,185	21,719	863	590
JPHC II	Japanese residents of 6 public health center areas in Japan	40–69	1993–1994	78,825	80%	Cancer registry and death certificate	40–69	2013	17.1	28,980	32,152	1,015	663
JACC	Residents from 45 areas throughout Japan	40–79	1988–1990	110,585	83%	Cancer registry and death certificate	40–79	2009	13.1	24,921	35,925	639	492
MIYAGI	Residents of 14 municipalities in Miyagi Prefecture, Japan	40–64	1990	47,605	92%	Cancer registry and death certificate	40–64	2014	21.3	21,174	22,751	1,059	703
OHSAKI	Beneficiaries of National Health Insurance among residents of 14 municipalities in Miyagi Prefecture, Japan	40–79	1994	54,996	95%	Cancer registry and death certificate	40–79	2008	10.8	21,606	23,338	707	401
TAKAYAMA	Residents of Takayama, Gifu Prefecture, Japan	35–101	1992	31,552	92%	Cancer registry and death certificates	35–101	2008	13.6	13,653	15,854	430	330
3-pref MIYAGI	Residents of 3 municipalities in Miyagi Prefecture, Japan	40+	1984	31,345	94%	Cancer registry and death certificate	40+	1992	7.6	13,149	16,122	166	123
3-pref AICHI	Residents of 2 municipalities in Aichi Prefecture, Japan	40–103	1985	33,529	90%	Cancer registry and death certificate	40–103	2000	11.4	15,334	17,010	245	199
3-pref OSAKA	Residents of 4 municipalities in Osaka Prefecture, Japan	40–97	1983	35,755	82%	Cancer registry and death certificate	40–97	2000	12.2	16,036	18,098	250	176
LSS	Atomic bomb survivors in Hiroshima and Nagasaki, Japan	46–104	1991	13,591	100%	Cancer registry and death certificate	46–104	2003	10.7	4,506	7,550	199	220

Total				499,585						179,544	210,519	5,573	3,897

JACC, Japan Collaborative Cohort Study; JPHC, Japan Public Health Center-based prospective Study; LSS, Life Span Study; MIYAGI, Miyagi Cohort Study; OHSAKI, Ohsaki National Health Insurance Cohort Study; TAKAYAMA, Takayama Study; 3-pref MIYAGI, Three Prefecture Study – Miyagi portion; 3-pref AICHI, Three Prefecture Study – Aichi portion; 3-pref OSAKA, Three Prefecture Study – Osaka portion.

### Assessment of height

Participants self-reported their height at baseline. We categorized participants into five groups: <160 cm, 160–162 cm, 163–165 cm, 166–169 cm, and ≥170 cm for men and <148 cm, 148–150 cm, 151–153 cm, 154–156 cm, and ≥157 cm for women. In this categorization, we considered that each group should have a sufficient and equal number of participants, with reference to a previous study.^20^

### Case ascertainment

The participants were followed up for cancer incidence from the baseline survey (JPHC I, 1990; JPHC II, 1993–1994; JACC, 1988–1990; MIYAGI, 1990; OHSAKI, 1994; TAKAYAMA, 1992; 3-pref MIYAGI, 1984; 3-pref AICHI, 1985; 3-pref OSAKA, 1983; LSS, 1991) until the date of diagnosis of cancer, the date of emigration outside the study area, the date of death, or the date of end of follow-up in each cohort (JPHC I, 2013; JPHC II, 2013; JACC, 2009; MIYAGI, 2014; OHSAKI, 2008; TAKAYAMA, 2008; 3-pref MIYAGI, 1992; 3-pref AICHI, 2000; 3-pref OSAKA, 2000; LSS, 2003). The residential registry was used to confirm residence status, including survival. For deceased cases, the cause of death was determined from the date certificates and coded according to the International Classification of Diseases, Tenth Revision^21^ and for cancer cases according to the International Classification of Diseases for Oncology, Third Edition (ICD-O-3).^22^ The study outcomes were incident CRC (ICD-O-3, codes C18.0–18.9, C19, and C20); colon cancer (ICD-O-3, codes C18.0–18.9), including duplication in the colon (C18.8) and unknown site (C18.9); rectal cancer (ICD-O-3, codes C19.9, and C20.9); proximal colon cancer (PCC) (ICD-O-3, codes C18.0–18.5); and distal colon cancer (DCC) (ICD-O-3, codes C18.6–18.7) diagnosed during the respective follow-up periods of each study.

### Statistical analysis

In each cohort, person-years of follow-up were calculated from the date of baseline survey through the date of diagnosis or death due CRC, migration from the study area, death from any cause, or the end of follow-up, whichever occurred first. In the analysis for the cancer in a subsite of the colorectum, incidence of cancer in other subsites were censored. For instance, if rectal cancer was detected 2 years after colon cancer, we used information on colon cancer only. Incidence of cancer other than CRC was not treated as a censored case. All the analyses were done for men and women separately. The hazard ratios (HRs) and 95% confidence intervals (CIs) of CRC and its subsites were calculated using Cox proportional hazards regression for each category of height, with the lowest category serving as the reference. Each study estimated two types of HRs: age- (years, continuous) and area-adjusted (within each study for JPHC-I, JPHC-II, JACC, and LSS) HR and multivariable-adjusted HR. In addition to age and area adjustments, the multivariable-adjusted model included body mass index (14 to <18.5, 18.5 to <22, 22 to <25, 25 to <30, or 30 to <40 kg/m^2^), history of diabetes (yes or no), smoking status (for men, never smoker, past smoker, current smoker of <20, or ≥20 cigarettes/day; for women, never smoker, past smoker, or current smoker), alcohol drinking (for men, never/former drinker, occasional drinker of <once/week, or current drinker of <23, 23 to <46, 46 to <69, 69 to <92, or ≥92 ethanol g/day; for women, never/former drinker, occasional drinker of <once/week, or current drinker of <23 or ≥23 ethanol g/day), and leisure time sports or physical activity (JPHC I and JPHC II, almost never, 1–3 days/month, or ≥1 days/week; JACC, MIYAGI, and OHSAKI, almost never or ≥1 hour/week; TAKAYAMA, no, 1–2, or ≥2 hour/week; LSS: almost never, or ≥1 day/week), log-transformed energy intake (continuous), energy-adjusted dietary intake of red meat containing beef, pork and liver (quartiles), processed meat (quartiles), calcium (quartiles) or milk (LSS, 1–2 times/week or less, 3–4 times/week, almost every day), fiber (quartiles) or green and yellow vegetable (LSS, less than once a week, more than 2–4 times a week, almost every day). An indicator term for the missing data was created for each covariate. Sensitivity analysis was performed by excluding cases diagnosed within 3 years of the baseline. The trend association was assessed by calculating the regression coefficient and standard error of the linear trend, with ordinal numbers assigned to the five height categories. SAS (SAS Institute, Cary, NC, USA) or Stata (StataCorp, College Station, TX, USA) statistical software were used to perform all statistical analyses.

To obtain a summary estimate, we combined resulting HRs across the cohorts using a random-effects model.^23^ We selected this model considering the differences in the background of study population, study location, the timing of baseline survey across the participating cohorts. We did not include studies that had no cases for a category in the calculation of the pooled estimate for that category. We examined the trend association by combining the regression coefficients and standard errors of the linear trend across the participating cohorts using a random-effects model. Heterogeneity for each category was indicated by Cochran’s *Q*-statistic,^23^ which was considered statistically significant when the *P*-value was less than 0.10. The *I*^2^-statistic was reported to describe the percentage of the total variation in the study-specific HRs that was due to heterogeneity. We created forest plots to present the HRs of CRC, colon, and rectum for the highest versus lowest categories of height in each participating cohort and their summary estimates.

## RESULTS

Among 390,063 participants (179,544 men and 210,519 women) with 5,672,930 person-years of follow-up (2,559,144 men and 3,113,786 women), we identified 9,470 cases of CRC (5,573 men and 3,897 women) (Table 1). eTable 1 shows the mean and standard deviation, and interquartile range of height of participants in each study.

**Figure 1.**
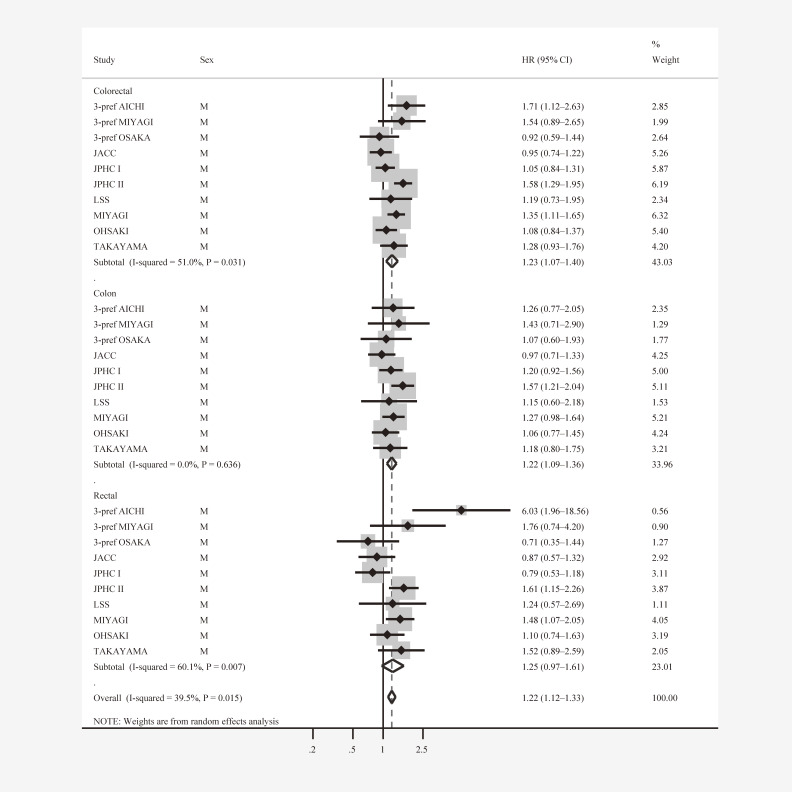
Height (highest vs lowest exposure category) and colorectal cancer subsites among men in ten Japanese cohort studies. CI, confidence interval; DL, DerSimonian-Laird; HR, hazard ratio: M, men. JACC, Japan Collaborative Cohort Study; JPHC, Japan Public Health Center-based prospective Study; LSS, Life Span Study; MIYAGI, Miyagi Cohort Study; OHSAKI, Ohsaki National Health Insurance Cohort Study; TAKAYAMA, Takayama Study; 3-pref MIYAGI, Three Prefecture Study – Miyagi portion; 3-pref AICHI, Three Prefecture Study – Aichi portion; 3-pref OSAKA, Three Prefecture Study – Osaka portion.

Table 2 shows the pooled estimates for men. We observed a statistically significant association between height and CRC and colon cancer risk: multivariable-adjusted HR for the highest category of height “≥170” versus lowest category “<160 cm” was 1.23 (95% CI, 1.07–1.40) for CRC and 1.22 (95% CI, 1.09–1.36) for colon cancer. The association was virtually unchanged after excluding cases diagnosed within 3 years of baseline for CRC and colon cancer. CRC and colon cancer risk increased by 6% for each 5 cm increment in height. For colon subsites, there was a significant association between height and DCC (HR 1.27; 95% CI, 1.08–1.49), and the risk of DCC was increased by 7% for a 5 cm increment in height. In contrast, no such association was observed for PCC (HR 1.08; 95% CI, 0.86–1.36). The risk of rectal cancer for the highest versus lowest height group was increased (HR 1.25; 95% CI, 0.97–1.61), and this association was strengthened and became statistically significant after excluding cases diagnosed during the first 3 years of follow-up (HR 1.31; 95% CI, 1.01–1.70). The test for heterogeneity across studies was statistically significant in CRC and rectal cancer cases but not for colon cancer, PCC, and DCC (Table 2 and Figure 1).

**Table 2.** Pooled multivariable hazard ratios and 95% confidence intervals for the association between height and colorectal cancer in men

	Total	Height categories					Trend (per 5 cm)	*P* for trend	Heterogeneity^b^ P, I^b^ (%)

		<160 cm	160–162 cm	163–165 cm	166–169 cm	≥170 cm			
Number of subjects	179,544	42,109	31,519	40,890	29,129	35,897			
Person-years	2,559,144	568,744	452,566	588,374	426,984	522,476			
**Colorectal cancer**									
Number of colorectal cancer cases	5,573	1,351	1,020	1,289	903	1,010			
Age- and area-adjusted HR (95% CI)		1.00 (Ref.)	**1.11 (1.02–1.21)**	**1.17 (1.06–1.29)**	**1.28 (1.14–1.44)**	**1.26 (1.10–1.44)**	**1.07 (1.03–1.11)**	**0.001**	0.03, 51.3
Multivariable-adjusted HR^a^ (95% CI)		1.00 (Ref.)	1.10 (0.98–1.20)	**1.16 (1.05–1.27)**	**1.25 (1.11–1.40)**	**1.23 (1.07–1.40)**	**1.06 (1.02–1.10)**	**0.002**	0.03, 51.0
Multivariable-adjusted HR excluding cases ​ <3 years^a^ (95% CI)		1.00 (Ref.)	**1.12 (1.00–1.24)**	**1.17 (1.04–1.33)**	**1.25 (1.09–1.43)**	**1.24 (1.08–1.42)**	**1.06 (1.02–1.11)**	**0.004**	0.07, 44.0

**Colon cancer**									
Number of colon cancer cases	3,621	878	656	862	594	631			
Age- and area-adjusted HR (95% CI)		1.00 (Ref.)	1.10 (0.99–1.23)	**1.21 (1.09–1.36)**	**1.29 (1.16–1.44)**	**1.25 (1.12–1.40)**	**1.07 (1.03–1.10)**	**<0.001**	0.58, 0.0
Multivariable-adjusted HR^a^ (95% CI)		1.00 (Ref.)	1.09 (0.98–1.21)	**1.20 (1.07–1.34)**	**1.25 (1.12–1.41)**	**1.22 (1.09–1.36)**	**1.06 (1.03–1.09)**	**<0.001**	0.64, 0.0
Multivariable-adjusted HR excluding cases ​ <3 years^a^ (95% CI)		1.00 (Ref.)	1.10 (0.97–1.24)	**1.19 (1.03–1.38)**	**1.23 (1.07–1.40)**	**1.21 (1.08–1.36)**	**1.06 (1.02–1.10)**	**0.003**	0.45, 0.0

**Proximal colon cancer**									
Number of proximal colon cancer cases	1,307	364	270	329	238	226			
Age- and area-adjusted HR (95% CI)		1.00 (Ref.)	1.10 (0.93–1.30)	1.13 (0.90–1.40)	**1.36 (1.07–1.72)**	1.10 (0.88–1.39)	1.04 (0.98–1.11)	0.168	0.16, 30.6
Multivariable-adjusted HR^a^ (95% CI)		1.00 (Ref.)	1.09 (0.92–1.30)	1.11 (0.89–1.39)	**1.32 (1.04–1.68)**	1.08 (0.86–1.36)	1.04 (0.97–1.10)	0.257	0.18, 28.5
Multivariable-adjusted HR excluding cases ​ <3 years^a^ (95% CI)		1.00 (Ref.)	1.12 (0.91–1.36)	1.08 (0.83–1.41)	1.26 (0.99–1.61)	1.00 (0.74–1.34)	1.03 (0.96–1.10)	0.489	0.05, 46.4

**Distal colon cancer**									
Number of distal colon cancer cases	1,557	398	291	407	295	326			
Age- and area-adjusted HR (95% CI)		1.00 (Ref.)	1.05 (0.90–1.22)	**1.23 (1.02–1.48)**	**1.33 (1.13–1.55)**	**1.32 (1.13–1.54)**	**1.08 (1.03–1.13)**	**0.001**	0.60, 0.0
Multivariable-adjusted HR^a^ (95% CI)		1.00 (Ref.)	1.03 (0.88–1.20)	**1.21 (1.00–1.45)**	**1.26 (1.08–1.48)**	**1.27 (1.08–1.49)**	**1.07 (1.03–1.11)**	**0.001**	0.67, 0.0
Multivariable-adjusted HR excluding cases ​ <3 years^a^ (95% CI)		1.00 (Ref.)	1.03 (0.87–1.22)	1.21 (0.99–1.48)	**1.25 (1.05–1.49)**	**1.32 (1.12–1.57)**	**1.07 (1.04–1.11)**	**<0.001**	0.85, 0.0

**Rectal cancer**									
Number of rectal cancer cases	1,973	480	368	432	313	380			
Age- and area-adjusted HR (95% CI)		1.00 (Ref.)	1.12 (0.97–1.29)	1.09 (0.90–1.32)	1.24 (0.95–1.62)	**1.28 (1.00–1.64)**	1.05 (0.99–1.13)	0.117	0.01, 58.7
Multivariable-adjusted HR^a^ (95% CI)		1.00 (Ref.)	1.10 (0.95–1.28)	1.08 (0.89–1.31)	1.21 (0.92–1.60)	1.25 (0.97–1.61)	1.05 (0.98–1.12)	0.117	0.01, 60.1
Multivariable-adjusted HR excluding cases ​ <3 years^a^ (95% CI)		1.00 (Ref.)	1.15 (0.99–1.35)	1.08 (0.89–1.31)	1.26 (0.95–1.67)	**1.31 (1.01–1.70)**	1.07 (0.99–1.15)	0.098	0.02, 54.1

CI, confidence interval; HR, hazard ratio; JACC, Japan Collaborative Cohort Study; JPHC, Japan Public Health Center-based prospective Study; LSS, Life Span Study; MIYAGI, Miyagi Cohort Study; OHSAKI, Ohsaki National Health Insurance Cohort Study; TAKAYAMA, Takayama Study; 3-pref MIYAGI, Three Prefecture Study – Miyagi portion; 3-pref AICHI, Three Prefecture Study – Aichi portion; 3-pref OSAKA, Three Prefecture Study – Osaka portion.

^a^Adjusted for age, area, history of diabetes (yes or no), body mass index (14 to <18.5, 18.5 to <22, 22 to <25, 25 to <30, or 30 to <40 kg/m^2^), smoking status (for men: never smoker, past smoker, current smoker of 1–19 cigarettes/day, or current smoker of ≥20 cigarettes/day; for women: never smoker, past smoker, or current smoker), alcoholic drinking (for men: never/former drinker, occasional drinker of less than once a week, or current drinker of <23, 23 to <46, 46 to <69, 69 to <92, or ≥92 ethanol g/day; for women: never/former drinker, occasional drinker of less than once a week, or current drinker of <23 or ≥23 ethanol g/day), non-occupational physical activity (JPHC I and JPHC II: almost never, 1–3 day/month, or ≥1 day/week; JACC, MIYAGI, and OHSAKI: almost never or ≥1 hour/week), log-transformed energy intake (continuous), energy-adjusted dietary intake of red meat intake containing beef, pork, and liver (quartiles), processed meat (quartiles), calcium (quartiles), and fiber (quartiles). The 3-pref MIYAGI, 3-pref AICHI, and 3-pref OSAKA studies, for which information on red meat, calcium, and fiber intake was not available, adjusted for all type of meat intake containing processed meat (never, 1–2 day/month, or 1–2 day/week), milk (never, 1–2 day/month, or 1–2 day/week), green and yellow-colored vegetable intake (never, 1–2 day/month, or 1–2 day/week) as substitutes.

^b^For the highest category.

**Figure 2.**
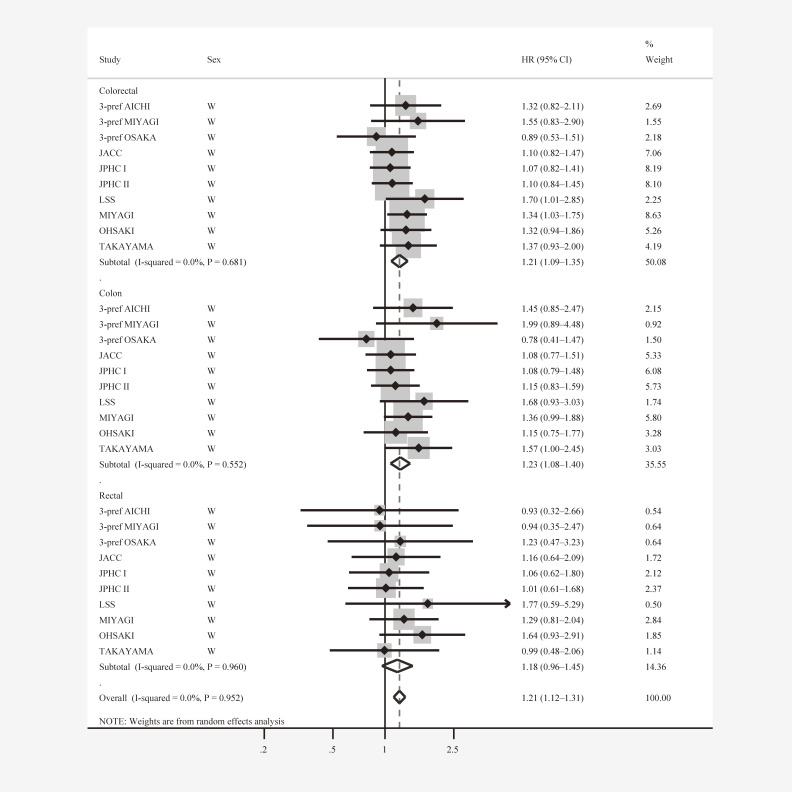
Height (highest vs lowest exposure category) and colorectal cancer subsites among women in ten Japanese cohort studies. CI, confidence interval; DL, DerSimonian-Laird; HR, hazard ratio: W, women. JACC, Japan Collaborative Cohort Study; JPHC, Japan Public Health Center-based prospective Study; LSS, Life Span Study; MIYAGI, Miyagi Cohort Study; OHSAKI, Ohsaki National Health Insurance Cohort Study; TAKAYAMA, Takayama Study; 3-pref MIYAGI, Three Prefecture Study – Miyagi portion; 3-pref AICHI, Three Prefecture Study – Aichi portion; 3-pref OSAKA, Three Prefecture Study – Osaka portion.

Table 3 shows the pooled estimates for women. Similar to the results in men, height was associated with a significant increase in CRC and colon cancer risk: multivariable-adjusted HR for the highest category of height “≥157” versus lowest category “<148 cm” was 1.21 (95% CI, 1.09–1.35) for CRC and 1.23 (95% CI, 1.08–1.40) for colon cancer. The association was virtually unchanged after excluding cases diagnosed within 3 years of the baseline. CRC and colon cancer risk increased by 5% and 6%, respectively, for every 5 cm increment in height. In the analysis by colon subsite, the association for DCC was statistically significant (HR 1.35; 95% CI, 1.003–1.81), and there was also a suggestion of an association for PCC (HR 1.19; 95% CI, 0.99–1.42). In the analysis of height as a continuous variable, PCC risk increased by 7% for each 5 cm increment in height (statistically significant), while in the case of DCC, the risk increased by 4% (statistically not significant). Regarding rectal cancer, there was an 18% increase, albeit not statistically significant, in risk associated with the highest stature (HR 1.18; 95% CI, 0.96–1.45). The test for heterogeneity across studies was not statistically significant in most analyses (Table 3 and Figure 2).

**Table 3.** Pooled multivariable hazard ratios and 95% confidence intervals for the association between height and colorectal cancer in women

	Total	Height categories					Trend (per 5 cm)	*P* for trend	Heterogeneity^b^ P, I^b^ (%)

		<148 cm	148–150 cm	151–153 cm	154–156 cm	≥157 cm			
Number of subjects	210,519	43,461	50,425	40,613	35,592	40,428			
Person-years	3,113,786	611,862	739,352	617,158	536,206	609,207			
**Colorectal cancer**									
Number of colorectal cancer cases	3,897	863	958	752	663	661			
Age- and area-adjusted HR (95% CI)		1.00 (Ref.)	1.06 (0.94–1.20)	**1.13 (1.02–1.25)**	**1.23 (1.10–1.37)**	**1.20 (1.08–1.34)**	**1.04 (1.01–1.07)**	**0.009**	0.74, 0.0
Multivariable-adjusted HR^a^ (95% CI)		1.00 (Ref.)	1.07 (0.95–1.21)	**1.14 (1.03–1.27)**	**1.24 (1.11–1.39)**	**1.21 (1.09–1.35)**	**1.05 (1.01–1.08)**	**0.007**	0.68, 0.0
Multivariable-adjusted HR excluding cases ​ <3 years^a^ (95% CI)		1.00 (Ref.)	1.06 (0.92–1.21)	**1.13 (1.02–1.27)**	**1.26 (1.12–1.41)**	**1.23 (1.09–1.38)**	**1.04 (1.01–1.08)**	**0.004**	0.88, 0.0

**Colon cancer**									
Number of colon cancer cases	2,767	639	688	537	445	458			
Age- and area-adjusted HR (95% CI)		1.00 (Ref.)	1.06 (0.93–1.21)	1.14 (0.99–1.31)	**1.17 (1.01–1.36)**	**1.20 (1.06–1.37)**	**1.05 (1.01–1.09)**	**0.011**	0.66, 0.0
Multivariable-adjusted HR^a^ (95% CI)		1.00 (Ref.)	1.08 (0.95–1.23)	**1.16 (1.00–1.33)**	**1.20 (1.03–1.39)**	**1.23 (1.08–1.40)**	**1.06 (1.02–1.10)**	**0.008**	0.55, 0.0
Multivariable-adjusted HR excluding cases ​ <3 years^a^ (95% CI)		1.00 (Ref.)	1.08 (0.93–1.24)	**1.18 (1.03–1.34)**	**1.25 (1.05–1.42)**	**1.24 (1.08–1.43)**	**1.06 (1.02–1.10)**	**0.004**	0.67, 0.0

**Proximal colon cancer**									
Number of proximal colon cancer cases	1,372	359	357	300	247	236			
Age- and area-adjusted HR (95% CI)		1.00 (Ref.)	0.99 (0.85–1.16)	1.15 (0.93–1.42)	1.20 (0.97–1.49)	1.16 (0.97–1.38)	**1.06 (1.01–1.12)**	**0.022**	0.56, 0.0
Multivariable-adjusted HR^a^ (95% CI)		1.00 (Ref.)	1.01 (0.86–1.17)	1.16 (0.93–1.44)	1.23 (0.98–1.53)	1.19 (0.99–1.42)	**1.07 (1.01–1.13)**	**0.016**	0.50, 0.0
Multivariable-adjusted HR excluding cases ​ <3 years^a^ (95% CI)		1.00 (Ref.)	1.04 (0.88–1.23)	1.22 (0.99–1.49)	**1.30 (1.05–1.61)**	1.23 (0.99–1.51)	**1.09 (1.03–1.15)**	**0.004**	0.35, 10.6

**Distal colon cancer^c^**									
Number of distal colon cancer cases	791	177	234	178	145	175			
Age- and area-adjusted HR (95% CI)		1.00 (Ref.)	1.17 (0.93–1.46)	1.19 (0.95–1.49)	1.18 (0.88–1.57)	**1.34 (1.10–1.78)**	1.04 (0.98–1.10)	0.164	0.16, 32.7
Multivariable-adjusted HR^a^ (95% CI)		1.00 (Ref.)	1.17 (0.93–1.48)	1.21 (0.96–1.54)	1.19 (0.88–1.60)	**1.35 (1.003–1.81)**	1.04 (0.99–1.10)	0.151	0.13, 35.5
Multivariable-adjusted HR excluding cases ​ <3 years^a^ (95% CI)		1.00 (Ref.)	1.13 (0.88–1.45)	1.13 (0.94–1.51)	1.15 (0.86–1.54)	**1.34 (1.00–1.78)**	1.03 (0.98–1.07)	0.219	0.23, 24.1

**Rectal cancer**									
Number of rectal cancer cases	1,137	225	272	216	221	203			
Age- and area-adjusted HR (95% CI)		1.00 (Ref.)	1.07 (0.89–1.28)	1.13 (0.93–1.37)	**1.38 (1.14–1.68)**	1.20 (0.98–1.47)	1.01 (0.98–1.05)	0.367	0.97, 0.0
Multivariable-adjusted HR^a^ (95% CI)		1.00 (Ref.)	1.07 (0.89–1.28)	1.12 (0.92–1.36)	**1.38 (1.13–1.67)**	1.18 (0.96–1.45)	1.01 (0.98–1.05)	0.424	0.96, 0.0
Multivariable-adjusted HR excluding cases ​ <3 years^a^ (95% CI)		1.00 (Ref.)	1.02 (0.84–1.25)	1.04 (0.84–1.29)	**1.36 (1.11–1.68)**	1.19 (0.95–1.48)	1.02 (0.98–1.05)	0.328	0.98, 0.0

CI, confidence interval; HR, hazard ratio; JACC, Japan Collaborative Cohort Study; JPHC, Japan Public Health Center-based prospective Study; LSS, Life Span Study; MIYAGI, Miyagi Cohort Study; OHSAKI, Ohsaki National Health Insurance Cohort Study; TAKAYAMA, Takayama Study; 3-pref MIYAGI, Three Prefecture Study – Miyagi portion; 3-pref AICHI, Three Prefecture Study – Aichi portion; 3-pref OSAKA, Three Prefecture Study – Osaka portion.

^a^Adjusted for age, area, history of diabetes (yes or no), body mass index (14 to <18.5, 18.5 to <22, 22 to <25, 25 to <30, or 30 to <40 kg/m^2^), smoking status (for men: never smoker, past smoker, current smoker of 1–19 cigarettes/day, or current smoker of ≥20 cigarettes/day; for women: never smoker, past smoker, or current smoker), alcoholic drinking (for men: never/former drinker, occasional drinker of less than once a week, or current drinker of <23, 23 to <46, 46 to <69, 69 to <92, or ≥92 ethanol g/day; for women: never/former drinker, occasional drinker of less than once a week, or current drinker of <23 or ≥23 ethanol g/day), non-occupational physical activity (JPHC I and JPHC II: almost never, 1–3 day/month, or ≥1 day/week; JACC, MIYAGI, and OHSAKI: almost never or ≥1 hour/week), log-transformed energy intake (continuous), energy-adjusted dietary intake of red meat intake containing beef, pork, and liver (quartiles), processed meat (quartiles), calcium (quartiles), and fiber (quartiles). The 3-pref MIYAGI, 3-pref AICHI, and 3-pref OSAKA studies, for which information on red meat, calcium, and fiber intake was not available, adjusted for all type of meat intake containing processed meat (never, 1–2 day/month, or 1–2 day/week), milk (never, 1–2 day/month, or 1–2 day/week), green and yellow-colored vegetable intake (never, 1–2 day/month, or 1–2 day/week) as substitutes.

^b^For the highest category.

^c^3-pref MIYAGI was not included in the analysis due to lack of data.

## DISCUSSION

In this pooled analysis of 390,063 participants and 9,470 CRC cases in 10 population-based cohorts in Japan, CRC and colon cancer risk increased with increasing height in men as well as women. Rectal cancer risk was also high among individuals with tall stature, but this association was not significant in women. The present study, including a large sample size, provided strong evidence from an Asian population to support the association between adult height and CRC risk.

We found a 23% and 21% increase in CRC risk for men and women, respectively, among individuals with tall stature compared with those with short stature. This finding agrees with that of a meta-analysis of the global population by Song et al^7^ (21% in men and 32% in women). The magnitude of association on a continuous scale (per 5 cm increase in height) in our study (6% and 5% increase in men and women, respectively) is also comparable to those of meta-analyses by Abar et al^24^ (4% increase in men as well as women) and by the World Cancer Research Fund^9^ (4% and 6% increase in men and women, respectively). Mendelian randomization studies, another line of research that uses genetic variant associated with height as an instrumental variable, reported higher risk of CRC among individuals carrying a higher number of height-increasing alleles.^25^^,^^26^ The present study, including 10 large cohorts and adjusted for potentially important confounders, not only confirmed the association between tall stature and CRC risk but also extended the evidence among the Japanese population, who have experienced a sharp increase in both CRC and height after World War II.

The meta-analysis by Song et al^7^ showed a stronger association with colon cancer than rectal cancer in men (28% vs 12%) and women (41% vs 19%). One potential explanation for the stronger association with colon cancer could be that the colon is more susceptible to the effects of insulin and insulin-like growth factor (IGF-1) than the rectum,^27^ which has a significant role in growth^28^ and cancer promotion.^29^ In the present study, however, the risk of colon cancer and rectal cancer for the highest versus lowest categories of height was similarly increased in both men (22% and 25%) and women (23% and 18%), although the dose-response was more evident for colon cancer than rectal cancer in both sexes. Our findings align with those of the Asia Pacific Cohort Studies Collaboration^30^; the risk of death due to colon cancer (7% per 2 cm increase in height) and rectal cancer (5% per 2 cm increase in height) was similarly increased with increasing height, while statistical significance was attained only for the colon cancer. Our findings, along with the Asia Pacific Cohort Studies Collaboration, showed a higher risk of cancer in both colon and rectum among taller individuals; however, the association was less evident in the case of the rectum.

As the subsites of the colon (PCC and DCC) differ in molecular, pathological, and clinical features,^31^ the association between adult height and cancer risk might also be different. We previously reported differential associations for colon subsites associated with meat intake^32^ and smoking.^33^ In the present study, the risk of DCC and PCC for the highest versus lowest categories of height was stronger for DCC than for PCC in both men (27% vs 8%) and women (35% vs 19%), suggesting a greater impact of height determinants in carcinogenesis of the distal colon than that of the proximal colon. However, a large prospective study among United Kingdom women reported no measurable difference in cancer risk associated with tall stature between distal and proximal colons (24% vs 28%).^34^ Due to the scarcity of evidence, further investigations are necessary to draw a conclusion.

Some mechanisms behind the height-CRC association have been suggested. First, growth hormone and IGF-1, which stimulate growth during childhood and adolescence,^28^ is known to play a significant role in cancer progression by inhibiting apoptosis and stimulating cell proliferation.^29^ Second, more than 100 genes associated with height are linked to carcinogenesis via the regulation of cell growth, division, differentiation, senescence, and programmed death of the cells.^35^ Lastly, taller people have larger and longer intestines with a greater number of cells, which might increase CRC risk by increasing chance of cell mutations.^36^^,^^37^

The present pooling study has several strengths. This study covered major population-based cohort studies across Japan. Each cohort included in this study analyzed data using the same category of height while adjusting for a common set of covariates that are known to influence CRC risk. With many study participants as well as CRC incident cases, we were able to examine the association between height and CRC and its subsites with reasonable statistical power. Nevertheless, some limitations of the study need to be mentioned. First, all cohorts included in this study assessed height based on self-report, which might have led to overreporting or underreporting. However, the accuracy of self-reported height is high among Japanese adults.^38^^,^^39^ Second, although we adjusted for established risk/protective factors for CRC, we cannot exclude a possibility of bias due to residual confounding. We did not adjust for socioeconomic factors (household income and education) that might affect both adult height and risk of CRC due to the lack of these data in some cohorts.

In conclusion, the present pooled analysis of 10 large cohort studies in Japan confirms an increase in the risk of CRC and colon cancer (especially DCC) among men and women with relatively tall stature. The association with rectal cancer was less evident in both sexes. This study adds evidence to support that adult height could be used to identify individuals at a higher risk of CRC.

## SUPPLEMENTARY MATERIAL

eTable 1. Demographic characteristics of the analytical cohort studies in the present pooled analysis
