# Supplementary material for: Adult Height and Risk of Colorectal Cancer: A Pooled Analysis of 10 Population-based Cohort Studies in Japan
Source: J Epidemiol. 2024 Feb 5;34(2):94–103. doi: 10.2188/jea.JE20220289 (PMC10751191; doi:10.2188/jea.JE20220289)
Supplement: Supplementary file 1 [file je-34-094-s001.pdf]

eTable 1. Demographic characteristics of the analytical cohort studies in the present pooled analysis

	Study									
**Characteristics**	**JPHC I**	**JPHC II**	**JACC**	**MIYAGI**	**OHSAKI**	**TAKAYAMA**	**3-pref MIYAGI**	**3-pref AICHI**	**3-pref OSAKA**	**LSS**
**Men, n**	20,185	28,980	24,921	21,174	21,606	13,653	13,149	15,334	16,036	3,893
Age, years, mean [SD]	49.5 [5.9]	53.1 [8.8]	57.7[10.2]	51.6 [7.6]	59.1 [10.6]	54.3 [12.2]	56.5 [11.1]	55.6[11.1]	55.6 [11.2]	59.9 [10.6]
Height, cm										
Mean [SD]	163.8 [6.2]	164.3 [6.5]	162.9[6.6]	164.1 [6.3]	163.1 [12.7]	164.5 [6.9]	162.9 [7.6]	164.2 [6.4]	163.7 [6.3]	164.7 [6.4]
Median	164	165	163	164.5	163	165	163.1	165	164	165
Interquartile range (25–75%)	(160–168)	(160–169)	(159–167)	(160–168)	(159–168)	(160–169)	(159.5–167.5)	(160–168)	(160–168)	(160–169)
BMI ≥25 kg/m^2, %	27.7	26.8	18.2	27.4	25.8	17.0	22.4	15.3	16.7	17.7
Current smoker, %	53	51.8	50.1	60.1	48.9	53.8	41.3	54.1	58.2	50.5
Current drinker (≥23 ethanol g/day), %	61.7	56.3	68.1	51.4	46.2	79.3	11.8	45.8	26.4	36.7
**Women, n**	21,719	32,152	35,925	22,751	23,338	15,854	16,122	17,010	18,098	7,431
Age, years, mean [SD]	49.6 [5.9]	53.4 [8.9]	57.8 [10.0]	52.1 [7.4]	60.5 [10.0]	55.2 [13.0]	57.2 [11.4]	56.6 [11.5]	56.5 [11.6]	64.7 [11.5]
Height, cm										
Mean [SD]	151.6 [5.4]	152.2 [5.8]	151.1[5.9]	152.3 [5.5]	151.6 [14.9]	151.9 [6.4]	151.3 [6.5]	151.9 [5.9]	151.9 [5.9]	152.5 [5.9]
Median	152	152	151	152	151	152	151	152	152	152
Interquartile range (25–75%)	(148–155)	(149–156)	(148–155)	(149–156)	(148–155)	(148–156)	(148–155)	(149–156)	(149–155)	(150–156)
BMI ≥25 kg/m^2, %	29.1	26.1	22.2	31.3	31.9	13.6	28.0	14.2	19.1	16.2
Current smoker, %	5.7	7.3	4.6	6.8	6.8	12.9	6.5	11.2	10.7	6.3
Current drinker (≥23 ethanol g/day), %	5.6	6.4	17.1	2.4	3.0	35.7	0.5	24.6	1.8	1.7

JACC, Japan Collaborative Cohort Study; JPHC, Japan Public Health Center-based prospective Study; LSS, Life Span Study; MIYAGI, Miyagi Cohort Study; OHSAKI, Ohsaki National Health Insurance Cohort Study; TAKAYAMA, Takayama Study; 3-pref MIYAGI, Three Prefecture Study – Miyagi portion; 3-pref AICHI, Three Prefecture Study – Aichi portion; 3-pref OSAKA, Three Prefecture Study – Osaka portion.
